# Deception

**Published:** 1871-03

**Authors:** 


### PART IV.

### Editorials, Reviews, Items and News.

### DECEPTION.

A lie is a device which will fit every implement with which Satan may choose to work. When the Savior represented him as a liar and the father of lies, he depicted him in that character which he most loves to assume. Falsehood is his element—deception his business. It is by this means that he has wrought and is still working the larger portion of his mischief among mankind. By this he engenders strife between neighbor and neighbor; by this he stirs up wars between nation and nation. By putting into men tongues of lying which deceive and enrage, he has filled the land with sorrow and bloodshed.

No one can estimate the amount of suffering which falsehood has entailed upon mankind. It was a lie that caused the fall from primeval innocence, and through all the tide of time that has since flown on, Falsehood, in a thousand forms, has yielded a large harvest of human woe. A lie to stain the character of a friend, must be born of a spirit as fiendish as it is cowardly, and the rebound of such a lie, from the innocent to the perpetrator, will point him out as an object of loathing and contempt, which, through life's long journey, will cling to him as the shirt of Nessus.

Every lie is intended to deceive, but there are many modes of deception practiced which are not usually termed lies. Men deceive and impose upon each other in the common transactions of trade, who would repel indignantly the charge of being liars. Ladies, we are sorry to say, often deceive, in the rounds of social intercourse, who are not esteemed untruthful. It often appears as if there were an emulation between the sexes in the matter of deception. There is, indeed, throughout all the ranks of society too much of a disposition to deceive—too much effort to seem just what we are not. We find this, from the waning beauty, whose cheeks blush with carmine, to the speculator, who tries to hide his fears of impending bankruptcy, under a show of blustery bravado. There are ten thousand forms of deception to be seen in Vanity Fair. Some are ridiculous, some disgraceful, and some pathetic, by reason of the necessity which forces an ingenious spirit to deceive. All these artifices of fashion and trade are lies, and felt to be so by those who practice them. The very fact that they resort to deception, is a confession of weakness—an admission that they dare not face the truth. Weak people are always false, not because they lack the hardihood to avow and practice the truth. Men choose cunning rather than candor, not because they deem it the more admirable or more efficient quality, but because it is more adapted to the feebleness of their moral constitutions.

A very common form of deception is the telling of a literal truth, which produces the same effect which a lie would do. Frequently this requires no small exercise of ingenuity, and it seldom pays for the effort. The aim is to deceive, and at the same time to relieve the conscience from the burden of a falsehood. But we doubt whether the veriest casuist ever succeeded in thinking a lie any the less false, because he gave it the garb of truth. We are inclined to think that no other kind of lie inflicts as great an injury on one's moral nature. There is in the double effort to deceive ones self and another, a sense of meanness which is experienced in an equal degree in the perpetration of few other crimes. Lying is a cowardly vice—the refuge of mean spirits that have not moral courage enough to be bold. We cannot conceive of a truly brave man—the pure gentleman—resorting to falsehood. There is that in him so foreign to all that is false, that were he once to attempt playing the liar, he would be detected in the very first act.

Is it ever right to deceive? That we should not practice deception for our own benefit, admits of no question. But may we not deceive for the sake of the deceived party, or for some other good end? May not the physician beguile his patient with hopes which he knows to be false, if he thinks he may thereby prolong his life? May we conceal, by subterfuge and falsehood, one whom we believe innocent from the operations of an unjust law? These are questions often asked, and to which, we apprehend, there can be but one correct answer. No: we should speak and act the truth, *always*. The consequences are with God, and to us can be only matters of speculation. Our duty is clear and unequivocal. It is wrong and absurd for us to complicate it with results which may never take place, and for which, if they do, we are by no means responsible. We never can be sure that deception on our part would be of advantage to any other person, and if we were, it would still be our duty to stand to the right. We should not make our duty as to what *is*, yield to our notions as to what *may be*. If we may, for the sake of expediency, dispense with one article of the decalogue, why may we not with all? If we may lie in the cause of benevolence, why may we not steal and murder? If we begin to dispense with statutes whenever it suits our convenience, we will soon abrogate the whole law, and unseat Jehovah from His throne. If this result be bad, so is the beginning bad. The difference is in degree only, not in kind. To avoid that anarchy, we must stand unflinchingly to the letter and spirit of the law.

